# Mechanism Study on the Effects of Yellow River Sediment Silt Powder on Hydration, Microstructure, and Strength Development of Shotcrete

**DOI:** 10.3390/ma19112280

**Published:** 2026-05-28

**Authors:** Ge Zhang, Xin Wang, Jialing Li, Kunpeng Li, Yuanjian Wang, Ali Raza, Chengfang Yuan

**Affiliations:** 1 Yellow River Institute of Hydraulic Research, Yellow River Water Conservancy Commission, Zhengzhou 450003, China; 2 Key Laboratory of Yellow River, Ministry of Water Resources, Zhengzhou 450003, China; 3 School of Water Conservancy and Transportation, Zhengzhou University, Zhengzhou 450001, China; 4 College of Civil Engineering, Zhengzhou University, Zhengzhou 450001, China

**Keywords:** Yellow River sediment, shotcrete, hydration process, hydration products, microstructural evolution, strength development

## Abstract

To investigate the influence mechanism of Yellow River silt powder on the hydration process, microstructure, and strength development of shotcrete, and to promote the resource utilization of Yellow River sediment, this study systematically investigated the effects of different silt powder replacement levels (0%, 10%, 30%, and 50%) on a cement–accelerator system. A combination of setting time tests, isothermal calorimetry, and mechanical strength measurements was employed, together with microstructural characterization techniques including X-ray diffraction (XRD), Fourier transform infrared spectroscopy (FTIR), differential thermal analysis (DTA), and scanning electron microscopy (SEM). The results indicate that the silt powder content exerts a two-stage effect on the setting behavior of shotcrete. At low replacement levels (0–30%), both initial and final setting times are significantly prolonged, whereas at higher replacement levels (>30%), the setting time is anomalously shortened, approaching that of the reference mixture. The incorporation of silt powder delays the onset of the pre-induction period, prolongs the induction stage, and reduces the cumulative heat release, with the reduction exhibiting a staged trend characterized by gradual, pronounced, and then moderate changes as the replacement level increases. With increasing silt powder content, both compressive strength and splitting tensile strength decrease continuously. At a 50% replacement level, the 28-day compressive strength loss reaches 48.35%, while the splitting tensile strength loss reaches 43.30%, with more pronounced deterioration observed at early ages. The tensile-to-compressive strength ratio increases with silt powder content at early ages, while converging to similar values among all mixtures at later ages. Microstructural analysis indicates that silt powder primarily affects hydration through physical dilution and ion adsorption. At low dosages, nucleation effects slightly promote early hydration, whereas at high dosages, the hydration of calcium silicate phases is inhibited, resulting in reduced C–S–H gel formation and increased porosity. Additionally, AFt morphology transitions from dense prismatic crystals to loosely distributed needle-like structures. This study provides a systematic understanding of the role of silt powder in shotcrete and offers theoretical guidance for mix design optimization and the sustainable utilization of Yellow River sediment.

## 1. Introduction

Shotcrete is a type of concrete that is pneumatically projected at high velocity through a hose or pipeline onto a receiving surface, where it is immediately compacted upon impact [1,2]. Owing to its rapid setting, fast hydration and hardening, high early-age strength, and flexible construction process, shotcrete has been widely applied in railway, highway, hydraulic engineering, and mining projects [3,4]. In underground and slope stabilization engineering, shotcrete plays an essential role in providing immediate support and structural reinforcement [5,6]. Consequently, improving the performance and sustainability of shotcrete materials has become an important research topic in modern construction engineering [7,8]. In parallel with the demand for high-performance shotcrete, the sustainable utilization of natural resources has become increasingly critical [9,10]. The protection and sustainable management of the Yellow River is a critical national priority for the long-term development of China [11]. The Yellow River Basin is characterized by a unique hydrological condition with relatively low water discharge but extremely high sediment concentration [12,13]. The mismatch between water flow and sediment transport has resulted in severe sediment deposition throughout the basin [14]. Reservoir sedimentation has become a major technical challenge, particularly in flood control [15], maintaining effective reservoir storage capacity, and ensuring the safe and stable operation of irrigation systems [16]. Sediment deposition in reservoirs across different river basins in China shows significant variation [17,18]. Among them, the Yellow River Basin exhibits the most severe siltation, with reservoir storage capacity loss reaching approximately 26.82% [19]. At the same time, the demand for construction aggregates in China remains extremely high [20,21]. It is estimated that approximately 20 billion tons of sand and gravel are consumed annually for infrastructure development [22,23]. However, the excessive exploitation of natural river sand has led to resource depletion and environmental degradation, making the exploration of alternative materials increasingly necessary [24]. Therefore, utilizing sediment resources as construction materials presents a promising solution to both environmental and engineering challenges. In this context, Yellow River sediment, which is rich in fine particles and primarily composed of silicon dioxide (SiO_2_), has attracted growing attention as a potential substitute for conventional construction aggregates [25]. The effective utilization of Yellow River sediment not only alleviates the shortage of natural sand resources but also provides a sustainable pathway for sediment management and ecological protection [26]. Recent studies have explored the feasibility of incorporating Yellow River sediment into cement-based materials such as concrete and mortar [27,28]. The fine particle size and mineral composition of the sediment may influence the hydration behavior, microstructure development, and mechanical performance of cementitious systems [29,30]. In particular, the interaction between sediment particles and hydration products may alter the nucleation and growth of hydration phases, thereby affecting the overall performance of the material [31,32]. However, most existing studies focus primarily on conventional concrete systems [33], The incorporation of silt particles can induce complex changes in cement hydration kinetics and microstructural development [34,35]. Yellow River sediment differs significantly from conventional construction sand in terms of particle size distribution and physicochemical characteristics. It is predominantly composed of fine particles, mainly within the silt and clay size ranges (<0.075 mm), and typically exhibits flaky or angular particle shapes, rough surfaces and a relatively high clay mineral content. Owing to these characteristics, shotcrete incorporating Yellow River sediment may exhibit markedly different performance compared with shotcrete prepared with traditional river sand. Therefore, existing research findings cannot be directly applied to predict the behavior of Yellow River sediment-based shotcrete. In particular, when fine Yellow River sediment is used in shotcrete, its material compatibility and its effects on the setting and hardening behavior, mechanical properties, and microstructure of the cement–accelerator reaction system remain insufficiently understood. The extent of these effects and the underlying mechanisms also require systematic investigation and clarification.

Therefore, a comprehensive investigation combining macroscopic performance evaluation and microstructural characterization is required to clarify the mechanisms governing the influence of Yellow River sediment in shotcrete systems. Based on the above considerations, this study systematically investigates the effects of Yellow River silt powder on the hydration process, microstructure, and strength development of shotcrete. Different replacement levels of Yellow River silt powder were incorporated into a cement–accelerator system, and their influences on setting behavior, hydration heat evolution, and mechanical properties were evaluated. In addition, advanced microstructural characterization techniques, including X-ray diffraction (XRD), Fourier transform infrared spectroscopy (FTIR), differential thermal analysis (DTA), and scanning electron microscopy (SEM) were employed to reveal the underlying mechanisms. The findings of this research aim to provide a theoretical basis for the optimized utilization of Yellow River sediment in shotcrete and to promote the sustainable development of construction materials in sediment-rich regions.

## 2. Materials and Methods

### 2.1. Raw Material and Mixed Proportion

#### 2.1.1. Raw Material

**Figure 1:**
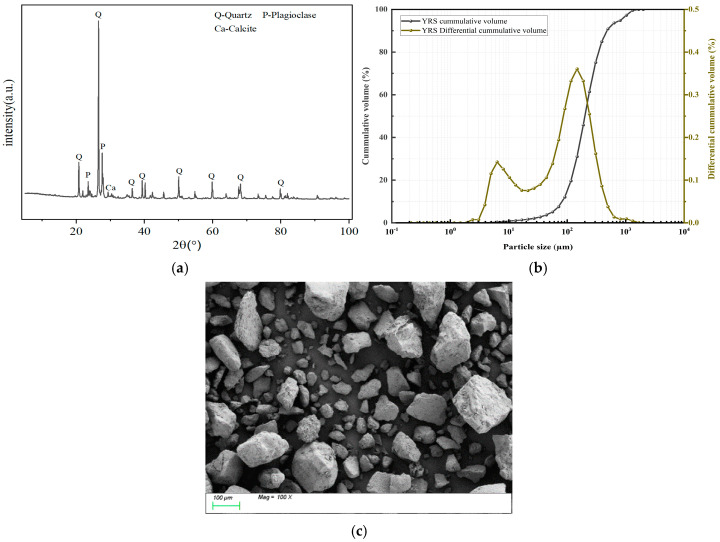
Physical and chemical properties of YRS. (**a**) YRS-XRD; (**b**) YRS particle size distribution; (**c**) YRS-SEM.

The raw materials used in this study primarily included cement, YRS, river sand, coarse aggregate, superplasticizer and accelerator. The YRS was sourced from the Xixiayuan Reservoir in Henan Province, initially in a moist state, and it was then dried before use; the main mineral compositions in the sediment include quartz, plagioclase, and calcium carbonate (Figure 1a). The median particle size of the YRS was determined to be 0.20 mm. Portland cement (P·I 42.5) conforming to the Chinese National Standard GB 8076 [36] (equivalent to CEM I 42.5) was used. The chemical compositions of the YRS and cement were analyzed using X-ray fluorescence (XRF), and the results are provided in Table 1. The particle sizes of the YRS were analyzed using a Malvern Mastersizer 2000 laser particle-size analyzer (Malvern Panalytical, Worcestershire, UK). The results are presented in Figure 1b, as shown in Figure 1b. SEM images shown in Figure 1c reveal that YRS particles display irregular geometric shapes, with considerable variation in particle size. River sand with a fineness modulus of 3.01, a bulk density of 2630 kg/m^3^, and a mud content of 0.2%. The coarse aggregate is single-grade limestone crushed stone with a size range of 5 to 10 mm, a bulk density of 2820 kg/m^3^, and a needle flake content of 3.7%. A polycarboxylate superplasticizer is selected for its water-reducing capabilities, with a water reduction rate of 30% and a solid content of 35%. The accelerators used SBT^®^-N (II) alkali-free liquid accelerator.

#### 2.1.2. Mixed Proportion

Two specimen preparation methods were adopted in this study. For the setting time, flowability, and microstructural tests, specimens were prepared under laboratory casting conditions. To systematically investigate the effect of fine-grained Yellow River sediment on material performance, the sediment was passed through a 200-mesh sieve to remove coarse-grained particles before use, and the resulting YRS silt powder was used to partially replace cement. The corresponding mix proportions are presented in Table 2. All specimens were prepared according to the designed mixing ratios. After mixing, the fresh paste was cast into molds and cured under standard conditions until the specified testing ages were reached.

For the strength tests, specimens were prepared by shotcrete spraying. Different from the laboratory-cast paste specimens, Yellow River sediment was used to partially replace river sand in the shotcrete mixtures, rather than using the YRS silt powder as a cement replacement. The corresponding mix proportions are shown in Table 3. The shotcrete was applied using the wet-mix spraying method. In accordance with the requirements for test specimen preparation specified in JGJ/T 372-2016 [37], Technical Specification for Application of Shotcrete, large panel molds with dimensions of 450 mm × 450 mm × 120 mm were used. After spraying, the large panel specimens were covered with plastic film for curing, kept at room temperature for one day, and then demolded. After demolding, the specimens were cut to the required dimensions using an infrared bridge-cutting machine. The cut specimens were subsequently placed in a standard curing room at 20 ± 2 °C and relative humidity above 95% until the designated testing ages.

### 2.2. Experimental Method

In this experiment, the effects of sediment powder dosage on the workability, mechanical properties, reaction progress, characteristic products, and microstructural properties of the matrix were analyzed. The analysis included setting time, compressive strength, splitting tensile strength, hydration heat, differential thermal analysis (DTA), x-ray diffraction (XRD), Fourier transform infrared spectroscopy (FTIR), and scanning electron microscopy (SEM). Table 4 presents the test grouping, including the size and number of specimens for each test.

#### 2.2.1. Setting Time Test

The setting time was tested according to GB/T 35159 (flash setting admixtures for shotcrete) [38]. The water-mineral admixture ratio used for determining the setting time of the paste was 0.35, and the average value was taken from three experiments in each group.

#### 2.2.2. Workability Test

The workability of the mixture was tested using a micro-collapse cylinder with an upper mouth of 50 mm, a lower mouth of 100 mm, a height of 150 mm, and a 60 cm × 60 cm plate.

#### 2.2.3. Strength Test

In order to fully evaluate the effect of YRS on the strength of the matrix, cube specimens with side lengths of 100 mm were used to test compressive strength and splitting tensile strength, following the requirements of GB/T 50081-2019, “Standard for Test Methods of Physical and Mechanical Properties of Concrete” [39]. The tensile-to-compressive strength ratio was calculated based on the test results.

#### 2.2.4. Fourier Transform Infrared Analysis

Fourier Transform Infrared (FTIR) spectroscopy was tested using a Shimadzu IRTracer 100 Fourier Transform Infrared Spectrometer (Shimadzu Corporation, Kyoto, Japan) with the following parameters: resolution of 4 cm^−1^, detector type of MIR TGS, and measurement range of 400–4000 cm^−1^ [40].

#### 2.2.5. X-Ray Diffraction Analysis

An X-ray diffractometer (Rigaku, Tokyo, Japan) was used for analysis. The samples were dried, ground, and then placed in a glass sample holder for testing. The scanning interval was 0.04° (2θ), the scanning speed was 2°/min, and the scanning range was 5–70° (2θ).

#### 2.2.6. Differential Thermal Analysis

A ZCT-B simultaneous thermal analyzer (Beijing Jingyi Hitech instrument Co., Ltd., Beijing, China) was employed, with the test sample weighing approximately 15 mg. The test was carried out under an argon atmosphere (flow rate: 50 mL/min) with a heating rate of 10 °C/min, programmed from 30 °C to 1000 °C. The DTA curves for each group of samples were obtained. Prior to testing, temperature calibration was performed using a standard substance (α-Al_2_O_3_), ensuring a temperature measurement accuracy of ±0.5 °C.

#### 2.2.7. Scanning Electron Microscopy Test

The microstructure of the samples was observed using a Sigma 300 field emission environmental scanning electron microscope (Carl Zeiss AG, Oberkochen, Germany). After curing to 28 days, the samples were broken with pliers, and hydration was stopped before testing [41].

## 3. Experiment Results and Analysis

### 3.1. Setting Time

**Figure 2:**
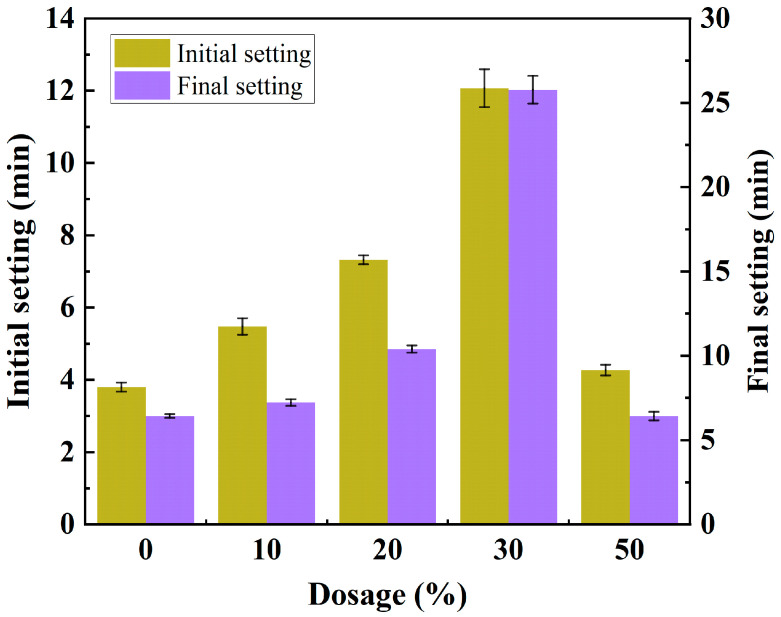
Influence of YRS silt powder content on setting time.

Figure 2 systematically investigates the effect of silt powder content on the setting time of cement paste. The results show that the incorporation of silt powder results in a distinct two-stage variation in setting behavior. At low replacement levels (0–30%), both the initial and final setting times increased markedly with increasing silt powder content. The initial setting time increased from 3.80 min for the reference mixture to 12.07 min at 30% replacement, equivalent to approximately 3.18 times that of the reference mixture. The final setting time increased from 6.43 min to 25.77 min, reaching approximately 4.01 times that of the reference mixture. This retardation is primarily attributed to the adsorption of Ca^2+^ and OH^−^ ions by silt particles (particle size range of 1–50 μm), which reduces the effective ionic concentration in the pore solution and delays the dissolution and precipitation processes of cement hydration. In addition, the relatively high Al_2_O_3_ content (approximately 18%) in the silt powder participates in the early hydration reactions, further modifying the reaction kinetics and delaying the formation of a continuous C–S–H gel network.

When the replacement level exceeds 30%, the setting time shows an abnormal reduction. At a replacement level of 50%, the initial setting time decreases sharply to 4.27 min, while the final setting time decreases to 6.42 min, approaching or even lower than that of the reference mixture. This transition can be attributed to several mechanisms. First, at high replacement levels, silt particles form a denser particle packing structure, which enhances water redistribution and accelerates ionic transport within the pore solution. Second, the release of soluble alkalis (total K^+^ and Na^+^ content of approximately 1.8%) increases the alkalinity of the pore solution, thereby accelerating the hydration kinetics. Third, XRD results indicate that reactive SiO_2_ in the silt powder reacts with Ca(OH)_2_ to form additional secondary hydration products, which contribute to the rapid development of rigidity in the system. In addition, the presence of the accelerator further promotes early hydration, amplifying the reduction in setting time at high replacement levels. However, silt powder exhibits a dual effect on the setting behavior of the cement–accelerator system, acting as a retarding component at low replacement levels and contributing to accelerated setting at higher replacement levels. This behavior provides important guidance for optimizing the dosage of silt powder in shotcrete applications.

### 3.2. Workability

**Figure 3:**
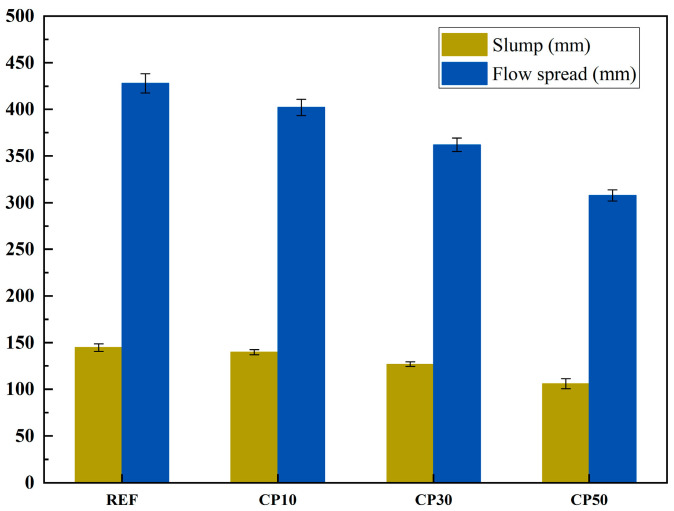
Effect of YRS silt powder content on workability.

Figure 3 presents the effect of silt powder content on the workability of the mixture. As shown, both slump and flow spread decrease with increasing silt powder content. The reference mixture exhibits a slump of 145 mm and a flow spread of 428 mm. When the replacement levels increase to 10%, 30%, and 50%, the slump decreases to 140 mm, 117 mm, and 90 mm, respectively, while the corresponding flow spread values are 402 mm, 370 mm, and 320 mm. Compared with the reference mixture, the slump decreases by 3.45%, 12.41%, and 26.90%, respectively, and the flow spread decreases by 6.07%, 15.42%, and 28.04%. These results indicate that increasing silt powder content significantly deteriorates the workability of the mixture. This effect becomes more pronounced when the replacement level exceeds 10%, where a substantial reduction in flowability is observed. The reduction in workability can be attributed to the strong adsorption capacity of silt particles, which absorb a large amount of free water from the cement paste, thereby reducing the effective liquid phase available for particle lubrication.

In addition, silt particles are predominantly flaky or needle-like inert particles, which disrupt the particle size distribution of the cement system and increase interparticle friction. When the replacement level exceeds 10%, the combined effects of water adsorption and unfavorable particle morphology lead to a continuous reduction of the lubrication layer within the paste, resulting in accelerated deterioration of workability.

### 3.3. Reaction Process

**Figure 4:**
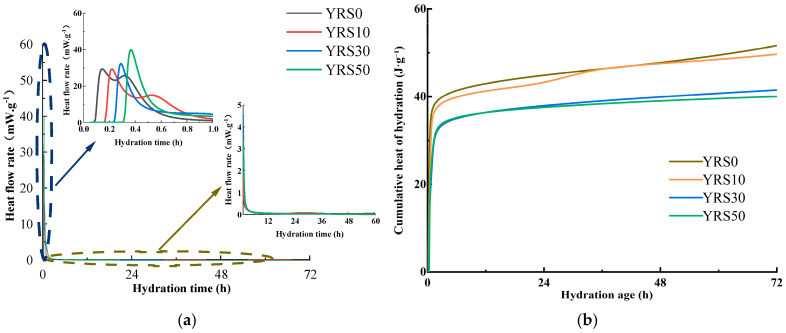
Effect of YRS silt powder content on hydration process. (**a**) Heat release rate; (**b**) cumulative heat of hydration.

Figure 4 illustrates the effect of silt powder content on the hydration kinetics of the cement-based system. Figure 4a presents the evolution of heat release rate, while Figure 4b shows the cumulative heat of hydration. As shown in Figure 4a, the incorporation of silt powder (0%, 10%, 30%, and 50%) significantly modifies the hydration process, exhibiting two distinct features. First, the onset of the pre-induction period is progressively delayed (0.09 h, 0.16 h, 0.24 h, and 0.31 h), while the corresponding heat release peak increases (29.3 mW/g, 29.3 mW/g, 32.2 mW/g, and 39.5 mW/g). This behavior is primarily attributed to the interaction between reactive Al_2_O_3_ (approximately 15–20%) in the silt powder and Ca^2+^ ions in the pore solution, together with the provision of additional heterogeneous nucleation sites by fine particles, which locally intensifies early-stage reactions. Second, the duration of the induction period is extended by approximately 40–60%, which can be ascribed to the preferential adsorption of Ca^2+^ and OH^−^ ions on silt particle surfaces.

This adsorption enhances the stability and thickness of the protective layer on C_3_S, thereby delaying its dissolution and subsequent hydration [42]. With increasing silt powder content, the acceleration stage becomes shorter and the peak heat release rate decreases. This trend is governed by several coupled mechanisms. The dilution effect reduces the volumetric content of reactive clinker phases, particularly C_3_S, thereby lowering the overall heat evolution. In addition, the heterogeneous and discontinuous precipitation of hydration products on silt particle surfaces restricts ionic diffusion and impedes the growth of hydration products. Furthermore, the gradual release of soluble alkalis (K^+^ and Na^+^) alters the pore solution chemistry and modifies the nucleation and growth kinetics of C–S–H gel. The presence of the accelerator further interacts with these processes, amplifying the sensitivity of early hydration kinetics to silt powder content. As shown in Figure 4b, the majority of heat release occurs within the first 24 h. The cumulative heat at 24 h for the reference, 10%, 30%, and 50% mixtures accounts for 87%, 87%, 91%, and 94% of the corresponding 72 h values, respectively, indicating a more concentrated early-age heat evolution with increasing silt powder content. Quantitatively, the cumulative heat at 24 h decreases from 44.9 J/g for the reference mixture to 43.3 J/g, 37.9 J/g, and 37.6 J/g for the 10%, 30%, and 50% mixtures, corresponding to reductions of 3.6%, 15.5%, and 16.3%, respectively.

The total heat release at 72 h further confirms this decreasing trend. The reference mixture exhibits a cumulative heat of 51.7 J/g, whereas the values for the 10%, 30%, and 50% mixtures are 49.7 J/g, 41.5 J/g, and 40.0 J/g, representing reductions of 3.8%, 19.6%, and 22.5%, respectively. The reduction in cumulative heat release with increasing silt powder content is mainly attributed to the combined effects of cement clinker dilution, adsorption of key ions (Ca^2+^ and OH^−^), and the formation of relatively low-density C–S–H gel at the silt–cement interface, which collectively reduce the overall hydration efficiency and matrix compactness. It is noteworthy that when the silt powder content exceeds 30%, the rate of reduction in cumulative heat becomes less pronounced, suggesting the existence of a critical threshold beyond which the influence of silt powder on hydration kinetics tends to stabilize. Overall, the incorporation of silt powder induces a nonlinear, staged reduction in hydration heat. When the replacement level is ≤10%, the reduction is limited (approximately 3–4%). In the range of 10–30%, a pronounced decrease is observed (15–20%), while beyond 30%, the variation becomes relatively moderate. This trend is consistent with the evolution of compressive strength. Combined XRD and SEM results indicate that, although higher silt powder contents further reduce total heat release, the adverse effect on strength development is partially mitigated by the micro-filling effect and improved particle packing. In addition, differential thermal analysis suggests that the delayed pozzolanic reaction of silt powder at later ages contributes to the gradual compensation of early-age hydration deficiency. These results demonstrate that silt powder exerts a coupled physicochemical effect on hydration kinetics, involving dilution, ion adsorption, nucleation, and micro-filling mechanisms, which collectively govern the heat evolution and subsequent performance of the cement-based system.

### 3.4. Strength Properties Results

#### 3.4.1. Compressive Strength

**Figure 5:**
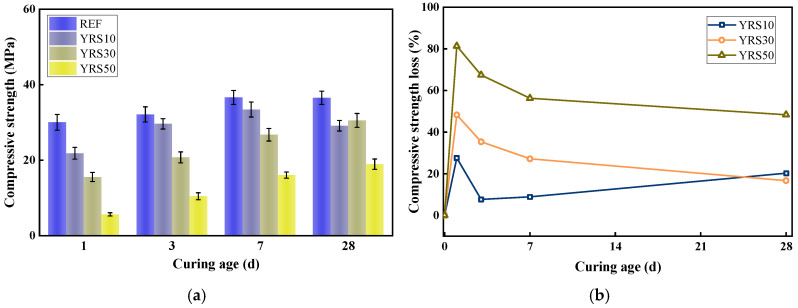
Effect of YRS content on the compressive strength of shotcrete. (**a**) Compressive strength; (**b**) compressive strength loss ratio.

Figure 5 illustrates the effect of YRS content on the compressive strength of hardened cement paste, where replacement levels of 10%, 30%, and 50% are denoted as YRS10, YRS30, and YRS50, respectively, and the mixture without YRS is defined as the reference (REF). As observed, the compressive strength decreases significantly with increasing YRS content. At 1 day, YRS10, YRS30, and YRS50 exhibit compressive strengths of 21.8 Mpa, 15.5 Mpa, and 5.6 Mpa, corresponding to reductions of 27.60%, 48.29%, and 81.36%, respectively, indicating that early-age strength deterioration becomes particularly severe when the replacement level exceeds 30%. Although all mixtures demonstrate continuous strength development with curing age, the strength disparity persists, and at 28 days, YRS10, YRS30, and YRS50 remain significantly lower than REF. This behavior can be attributed to multiple interacting mechanisms. Primarily, the dilution effect reduces the effective cement content, limiting the formation of load-bearing C–S–H gel and decreasing the degree of hydration. In addition, YRS particles may adsorb free water on their surfaces, reducing the effective water available for hydration and thereby slowing reaction kinetics. The presence of fine particles also alters particle size distribution and packing characteristics; while limited filling may occur at low contents, excessive YRS disrupts optimal packing density, leading to increased capillary porosity and pore connectivity. Furthermore, the interfacial transition zone (ITZ) surrounding YRS particles tends to be more porous and less cohesive due to weak physicochemical bonding, resulting in stress concentration under compressive loading. Another important factor is that unreactive or weakly reactive mineral phases in YRS may act as defects within the matrix, interrupting the continuity of the hydration network and reducing stiffness. Additionally, restrained hydration space and internal curing imbalance may lead to non-uniform hydration product distribution, further aggravating structural weakness. Although a slight nucleation effect may enhance early hydration at very low contents, this contribution is negligible compared to the dominant dilution and microstructural deterioration effects at higher replacement levels.

#### 3.4.2. Splitting Tensile Strength

**Figure 6:**
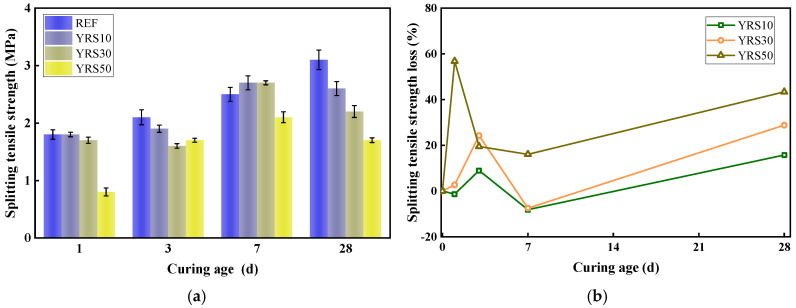
Effect of YRS content on splitting tensile strength of shotcrete. (**a**) Splitting tensile strength; (**b**) splitting tensile strength loss ratio.

Figure 6 presents the variation in splitting tensile strength and corresponding strength loss ratios with YRS content. The results reveal a clear stage-dependent reduction trend, with a particularly sharp decline observed at high replacement levels. At 1 day, YRS50 exhibits a drastic reduction of 56.76%, indicating that early-age tensile resistance is highly sensitive to the incorporation of YRS. At later ages, although all mixtures show strength development, the reductions remain significant, especially for YRS30 and YRS50. This pronounced sensitivity of tensile strength can be explained by fracture mechanics considerations, as tensile failure is governed by crack initiation and propagation rather than bulk resistance. The incorporation of silt powder introduces additional micro-voids and weak zones that act as preferential crack initiation sites, thereby reducing fracture energy. Moreover, the weak ITZ around YRS particles impairs stress transfer across the matrix, leading to premature crack coalescence under tensile loading. The presence of impurities such as YRS minerals may further hinder proper crystallization of hydration products, resulting in poorly bonded microstructures and reduced cohesion. In addition, differential shrinkage between YRS particles and the cement matrix can induce micro-cracking during drying and hydration, which accumulates over time and exacerbates tensile strength loss. Another contributing factor is the reduced formation of bridging hydration products, which are essential for resisting crack opening. As curing progresses, these defects become more interconnected, leading to a cumulative deterioration of tensile performance. Therefore, the negative impact of YRS on splitting tensile strength is not only due to reduced hydration but also strongly related to microstructural discontinuities and fracture-related mechanisms.

#### 3.4.3. Tension–Compression Ratio

**Figure 7:**
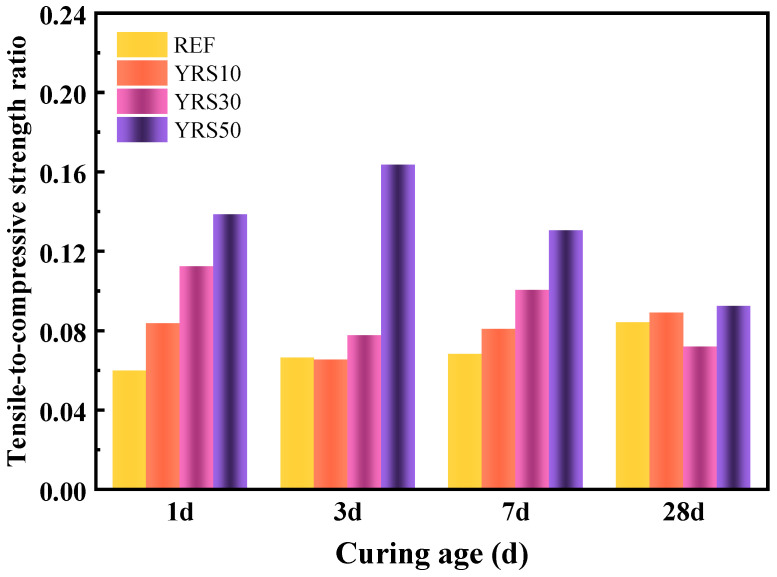
Effect of YRS content on tension–compression ratio of shotcrete.

Figure 7 illustrates the effect of sediment powder content on the tension–compression ratio of hardened cement paste. As observed, at early ages (within the first 3 days), all mixtures incorporating YRS exhibit higher tension–compression ratios than the reference (REF) mixture, with an overall increasing trend as the YRS content increases from YRS10 to YRS50. This behavior is primarily attributed to the role of YRS as a micro-filler at early stages, where its physical filling effect provides limited resistance to tensile cracking. However, its adverse impact on compressive strength development is more pronounced due to the reduction in effective hydration space and the formation of a relatively loose initial microstructure. Consequently, compressive strength is disproportionately weakened compared to splitting tensile strength, resulting in a temporary increase in the tension–compression ratio. In addition, the increased porosity and weaker load-bearing skeleton at early ages further amplify this difference. As curing progresses, continued hydration leads to gradual microstructural densification and improved bonding within the matrix, thereby reducing the disparity between tensile and compressive strengths. Accordingly, the differences in tension–compression ratio among the mixtures diminish over time. At 28 days, the ratios of all mixtures converge to comparable values, although YRS50 remains slightly higher than the other mixtures due to its persistently higher porosity, weaker interfacial transition zones, and lower overall compressive strength.

## 4. Analysis of Microstructural Mechanisms

### 4.1. Characteristic Hydration Products

**Figure 8:**
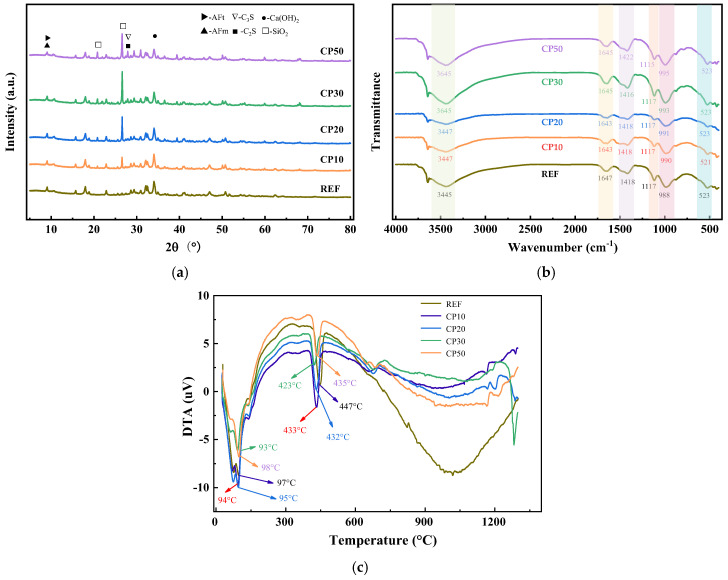
Effect of YRS silt powder content on characteristic hydration products of matrix after 28 days of standard curing. (**a**) XRD patterns; (**b**) FTIR spectra; (**c**) DTA curves.

The influence of YRS silt powder content on the hydration products of hardened cement paste was systematically investigated through multi-scale characterization techniques, including X-ray diffraction (XRD), Fourier-transform infrared spectroscopy (FTIR), and differential thermal analysis (DTA), as shown in Figure 8. The XRD results (Figure 8a) indicate that increasing YRS silt powder content does not significantly alter the characteristic diffraction peaks of Aft and Afm phases, suggesting that the fundamental crystalline structure of hydration products remains largely unchanged. However, the intensity of the SiO_2_ peak increases markedly with higher YRS silt powder content, confirming its predominantly inert nature. When the YRS silt powder content exceeds 30%, the diffraction peaks corresponding to unhydrated C_3_S and C_2_S become significantly more pronounced, while the Ca(OH)_2_ peak intensity decreases, indicating that the hydration of calcium silicate phases is substantially inhibited. This inhibition is strongly correlated with the observed compressive strength loss, suggesting that the reduced formation of hydration products is a primary factor governing strength deterioration at high replacement levels.

The FTIR analysis (Figure 8b) further shows that all samples exhibit consistent characteristic bands at 3640 cm^−1^ (Ca(OH)_2_), 1400 cm^−1^ (CO_3_^2−^), 1100 cm^−1^ (SO_4_^2−^), and 970 cm^−1^ (Si–O), while variations in peak intensity reflect differences in the quantity of hydration products rather than their chemical structure. This confirms that YRS silt powder primarily influences hydration through a physical dilution effect rather than chemical interaction. In addition, the presence of fine particles may restrict ion diffusion and reduce the availability of nucleation sites, thereby further limiting hydration kinetics. The DTA results (Figure 8c) reveal systematic variations in three characteristic temperature ranges: 80–100 °C (dehydration), 110–120 °C (C–S–H gel dehydration), and 400–500 °C (dihydroxylation). At low YRS silt powder content (<10%), the increased intensity of Aft and Ca(OH)_2_ decomposition peaks suggests that the microcrystalline nucleation effect of fine particles promotes early hydration. However, as the YRS silt powder content increases beyond 10–20%, the peak intensities decrease significantly, indicating a transition to a dilution-dominated regime. This reduction reflects decreased formation of hydration products, suppressed crystallization, and a lower degree of hydration. At low YRS silt powder contents (<10%), the increased intensities of the Aft- and Ca(OH)_2_-related decomposition peaks indicate that the fine particles may provide additional nucleation sites through a microcrystalline nucleation effect, thereby promoting early hydration. However, when the YRS silt powder content further increases to above 10–20%, the peak intensities decrease significantly, suggesting that the hydration process gradually shifts to a dilution-dominated regime. This decrease reflects a reduction in the formation of hydration products, inhibited crystallization, and a lower overall degree of hydration. In addition, a thermal decomposition peak is also observed at approximately 650 °C, which may be associated with the decarbonation of carbonate-containing products. These carbonate products may originate from two sources: on the one hand, the thermal decomposition of monocarboaluminate or hemicarboaluminate phases formed during hydration; on the other hand, the decomposition of CaCO_3_ present in the system. Overall, the DTA results further demonstrate that an appropriate amount of YRS silt powder can promote early hydration to some extent, whereas excessive incorporation weakens the hydration reaction and reduces the formation of major hydration products.

### 4.2. Matrix Microstructure

**Figure 9:**
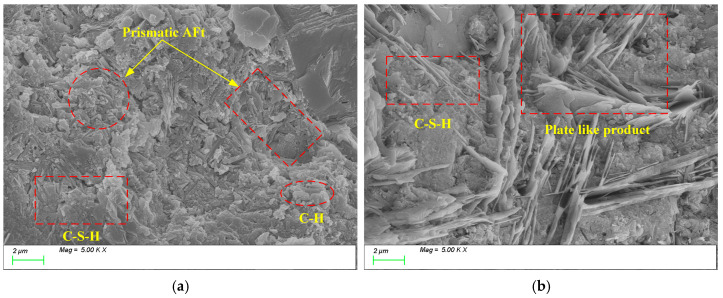

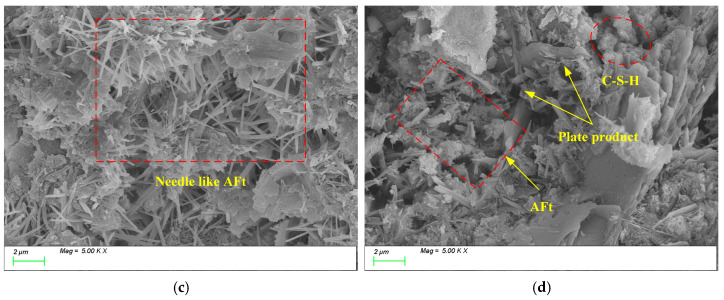
Effect of YRS silt powder content on microstructure of matrix after 28 days of standard curing. (**a**) REF; (**b**) CP10 (10% YRS silt powder); (**c**) CP30 (30% YRS silt powder); (**d**) CP50 (50% YRS silt powder).

Figure 9a presents the microstructure of the reference (REF) specimen after 28 days of hydration. A large number of prismatic Aft crystals can be clearly observed within the matrix, which is a typical morphological feature in cementitious systems incorporating accelerators. This phenomenon can be attributed to the fact that the addition of an accelerator significantly promotes the hydration of C_3_A phases while continuously supplying Al^3+^ and SO_4_^2−^ ions into the pore solution, thereby enhancing the nucleation and growth conditions of Aft. Unlike the slender needle-like Aft commonly observed in conventional cement systems, the Aft crystals in this case predominantly exhibit prismatic and short rod-like morphologies, indicating that the accelerator substantially modifies the crystallization behavior. In addition to the morphological transformation, the overall quantity of Aft is also noticeably increased. Furthermore, a considerable amount of dense and continuous flocculent C–S–H gel is formed within the matrix, exhibiting a layered and interwoven structure. This C–S–H gel is tightly integrated with hexagonal plate-like CH and prismatic Aft, forming a compact and well-connected microstructure. Such a dense microstructural network is considered a key factor contributing to the rapid early-age strength development and sustained long-term strength gain of the REF mixture. Figure 9b shows the microstructure of the CP10 specimen after 28 days of hydration. Compared with the REF mixture, the incorporation of 10% YRS silt powder results in noticeable changes in microstructural features. Specifically, YRS agglomerates and partially unreacted lamellar YRS particles are distributed within the matrix, exhibiting a layered stacking morphology. Notably, the C–S–H gel grows in a wrinkled or flocculent manner around these YRS particles and maintains a relatively dense and continuous interface, with no obvious microcracks or interfacial voids observed. This suggests that at low replacement levels, YRS silt powder can act as heterogeneous nucleation sites, promoting the localized growth of hydration products without significantly compromising matrix integrity. In addition, the lamellar structure of YRS particles may contribute to a micro-skeleton or filling effect, enhancing particle packing and supporting the spatial arrangement of C–S–H gel. Moreover, the fine particles may help refine pore structure by partially filling capillary voids, thereby improving local densification. These combined effects help explain why the compressive strength loss of CP10 remains relatively limited (within approximately 10%) across different curing ages.

This can be attributed to the dominant dilution effect, reduced hydration degree, and insufficient formation of binding phases. Furthermore, the excessive presence of clay particles may hinder ion diffusion and disrupt normal hydration product precipitation, leading to a heterogeneous and discontinuous microstructure. These factors collectively result in reduced load transfer efficiency and increased stress concentration, which explains the higher compressive strength loss observed in CP30. Figure 9d presents the microstructure of the CP50 specimen after 28 days of hydration. At this high YRS silt powder content, the microstructure exhibits severe degradation. The crystallization morphology of AFt is significantly altered, transforming from the relatively compact prismatic form observed in REF to a more dispersed and needle-like structure. This transformation can be attributed to several mechanisms: (i) YRS minerals in the silt powder adsorb substantial amounts of Ca^2+^ and SO_4_^2−^ ions, leading to ionic imbalance in the pore solution and promoting preferential crystal growth; (ii) the physical obstruction of silt powder particles disrupts the normal nucleation environment of AFt; and (iii) potential changes in local pH conditions affect crystallization kinetics. Although needle-like AFt possesses a higher specific surface area, its loose and discontinuous distribution results in a weaker structural framework. Additionally, the amount of C–S–H gel is significantly reduced and predominantly exhibits a flocculent morphology rather than the dense network observed in REF. The overall matrix becomes highly porous, with poor connectivity among hydration products and increased presence of large capillary voids. Moreover, the weak interfacial bonding between YRS particles and hydration products further exacerbates structural discontinuity. These microstructural deteriorations directly lead to a substantial reduction in matrix compactness and mechanical integrity, which explains the significantly lower compressive strength and the higher proportion of large pores (>1000 nm) observed in CP50.

## 5. Conclusions

This study systematically evaluated the influence of YRS silt powder on the setting behavior, hydration characteristics, mechanical performance, pore structure, and matrix microstructure of Yellow River sediment (YRS)-based shotcrete. The results reveal that the effect of sediment powder is governed by a distinct content-dependent mechanism, which can be summarized as follows.

(1) The effect of Yellow River sediment powder on setting time exhibits a pronounced stage-dependent behavior. At low replacement levels (0–30%), both the initial and final setting times are significantly prolonged with increasing sediment powder content. At a replacement level of 30%, the initial and final setting times are approximately 3.18 and 4.01 times those of the reference mixture, respectively. In contrast, when the replacement level further exceeds 30%, the setting time decreases markedly with increasing sediment powder content, and the setting behavior of the 50% replacement mixture approaches that of the reference mixture. The prolonged setting time at low replacement levels may be attributed to the adsorption of Ca^2+^ and OH^−^ ions by sediment particles, which reduces the effective ion concentration in the pore solution. Meanwhile, the active components in the sediment powder may interact with early hydration products, thereby delaying the formation of the C–S–H gel network. At higher replacement levels, the increased amount of sediment particles gradually forms a continuous packing skeleton, which facilitates the rapid establishment of the paste structure and consequently shortens the setting time.

(2) The incorporation of sediment powder significantly modifies the hydration process of shotcrete. Specifically, the presence of sediment delays the onset of the pre-induction period while increasing the corresponding heat evolution peak, prolongs the induction period by approximately 40–60%, and shortens the duration of the acceleration stage with a reduced peak heat flow rate. Furthermore, the cumulative heat release decreases progressively with increasing sediment content, with more pronounced reductions observed at 10–30% replacement levels, beyond which the rate of decrease stabilizes. This behavior indicates the existence of a critical threshold in sediment content, beyond which the influence on hydration kinetics transitions from reaction-controlled to dilution-dominated.

(3) In terms of mechanical performance, sediment powder exerts a significant adverse effect on both compressive strength and splitting tensile strength. The reduction in strength becomes particularly severe at high replacement levels (>30%), with the 50% mixture exhibiting a compressive strength loss of up to 81.36% at 1 day. Although strength continues to develop with curing age, the gap between mixtures remains substantial, and high-content mixtures still show pronounced strength deficiencies at 28 days. Additionally, the tension–compression ratio increases with sediment content at early ages, indicating that compressive strength is more severely affected than tensile strength during early hydration. As curing progresses, the ratios converge, reflecting gradual microstructural stabilization.

(4) The evolution of microstructure is identified as the fundamental mechanism governing the macroscopic performance of sediment-modified shotcrete. At low replacement levels, the YRS silt powder particles act as nucleation sites, promoting early hydration and facilitating the formation of a dense and well-bonded C–S–H matrix, particularly at 10% replacement. However, at higher replacement levels, the dilution effect dominates, suppressing the hydration of calcium silicate phases and increasing the content of unhydrated C_3_S and C_2_S while reducing Ca(OH)_2_ formation. Concurrently, the amount of C–S–H gel decreases and its structure becomes more loosely distributed, while AFt crystals transform from compact prismatic forms into dispersed needle-like morphologies. The matrix exhibits increased porosity, reduced connectivity of hydration products, and weakened interfacial bonding, ultimately leading to significant deterioration in mechanical properties.

(5) This study explains the coupled effects of Yellow River sediment powder on hydration kinetics, microstructural evolution, and strength development of shotcrete and identifies a critical content range governing performance transitions. The findings provide a theoretical basis for optimizing mixture design, controlling sediment content, and ensuring the engineering performance of sediment-based shotcrete, thereby contributing to the efficient and sustainable utilization of Yellow River sediment resources.

(6) Due to the limitations of the experimental conditions, this study mainly focused on the case in which the water dosage and accelerator dosage were kept constant. However, we acknowledge that the variation in the amount of active cementitious material and the accelerator-to-cement ratio may also influence the properties of the matrix. Therefore, in future work, additional control mixtures should be designed to maintain a constant accelerator-to-cement ratio and water-to-cement ratio so as to more rigorously isolate the effects of YRS incorporation. Alternatively, the experimental results should be normalized and discussed on the basis of cement mass, total binder mass, and/or paste volume to provide a more comprehensive evaluation of the influence of these variables on the matrix performance.

## Figures and Tables

**Table 1:** Chemical compositions of YRS and mineral admixtures (wt.%).

Minerals	SiO_2_	CaO	Al_2_O_3_	Fe_2_O_3_	K_2_O	TiO_2_	MgO	Other
YRS	68.64	8.40	12.33	3.25	2.55	0.74	2.05	2.04
Cement	22.64	64.88	4.68	3.57	—	—	2.94	1.29

**Table 2:** Mix proportions of different YRS silt powder contents.

No.	W/C	Cement	YRS Silt Powder	Accelerator	Water
REF	0.38	0.685	—	0.055	0.260
CP10	0.38	0.616	0.068	0.055	0.260
CP20	0.38	0.548	0.137	0.055	0.260
CP30	0.38	0.479	0.205	0.055	0.260
CP50	0.38	0.342	0.342	0.055	0.260

Note: CP stands for YRS silt powder. CP10 indicates a YRS silt powder content of 10%, and so on for the rest.

**Table 3:** Mix proportion of shotcrete (kg/m^3^).

No.	Cement	YRS	River Sand	Stone	Water	Water-Reducing Agent
REF	459	—	814	814	205.2	5.03
YRS10	459	81	733	810	205.2	5.03
YRS30	459	244	570	807	205.2	5.03
YRS50	459	407	407	800	205.2	5.03

YRS10 indicates that Yellow River sediment was used to replace river sand at a replacement ratio of 10%, and the rest is analogized.

**Table 4:** Grouping of the workability, mechanical, reaction progress, and microstructural property tests.

Properties	Performance Index	Specimen Size	Quantity
Setting time	Initial setting time	—	5
	final setting time	—	5
Workability	Slump	—	4
	Slump flow	—	4
Hydration heat	Heat evolution rate	—	4
	Accumulated hydration heat	—	4
Strength	Compressive strength	100 mm × 100 mm × 100 mm	48
	Splitting tensile strength	100 mm × 100 mm × 100 mm	48
Characteristic products	Differential thermal analysis	40 mm × 40 mm × 40 mm	15
	X-ray diffraction analysis Infrared spectroscopy analysis	40 mm × 40 mm × 40 mm	15
		40 mm × 40 mm × 40 mm	15
Microstructural properties	Scanning electron microscopy	40 mm × 40 mm × 40 mm	12

## Data Availability

The original contributions presented in the study are included in the article; further inquiries can be directed to the corresponding authors.
